# Supplementary material for: The Serum/Glucocorticoid-Regulated Kinase 1 Is Targeted by miR-19a in CD4+ T Cells
Source: Cells. 2022 Dec 29;12(1):133. doi: 10.3390/cells12010133 (PMC9818172; doi:10.3390/cells12010133)
Supplement: Supplementary file 1 [file cells-12-00133-s001.zip › cells-1987981-supplementary.pdf]

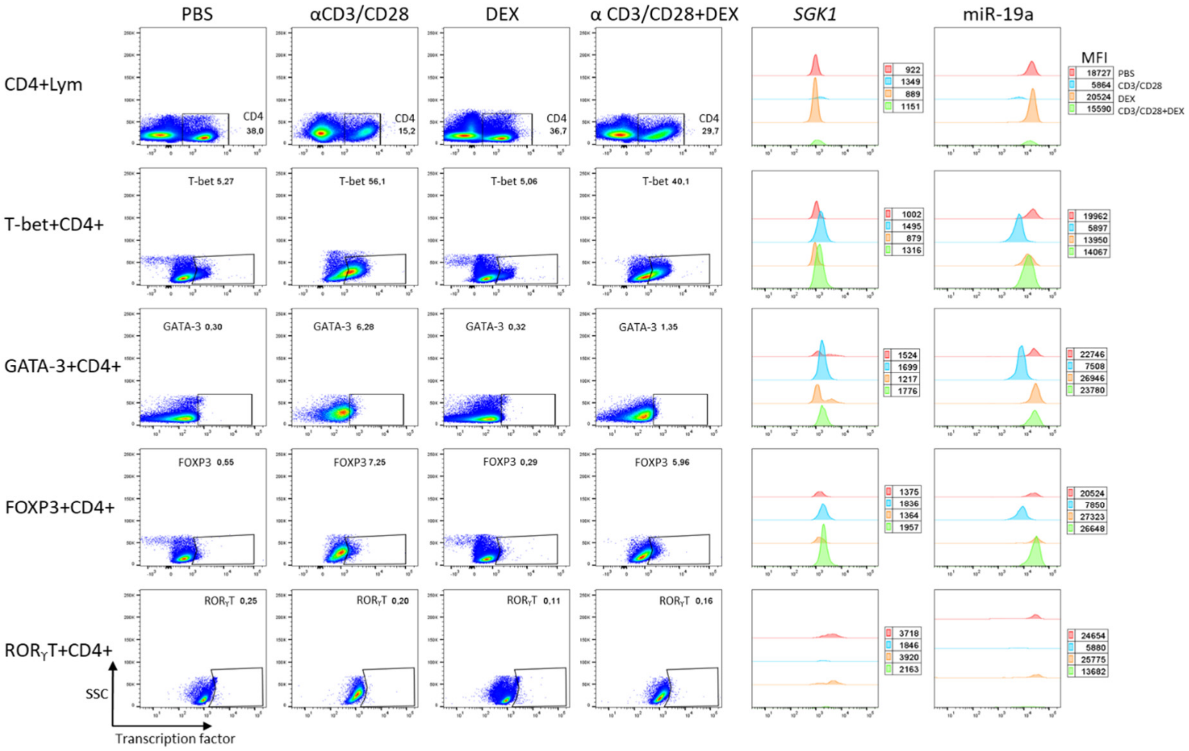

Figure S1. Gating strategy of PBMCs cultured for 48 hours followed by PrimeFlow RNA Assay. All flow cytometric analyses were performed with a BD FACSVerse Flow Cytometer running BD FACSuite Software (BDBiosciences, CA, USA). Mean number of acquired events was 1.1 x 10^6 for PrimeFlow RNA Assay and 0.9 x 10^6 for polarizing experiments. Data were analyzed with FlowJo Software (Tree Star Inc., OR, USA). Only lymphocytes determined as live by negative viability stain (Dead/Live aqua stain) and singlets were analyzed. CD4+ lymphocytes were considered TH cells. Transcription factor staining determining Th cell subclass were: TH1: T-bet+, TH2: GATA-3+, Treg: FOXP3+, TH17: RORγT. Antibodies used are found in Table S2. Target probe sets for *SGK1* and miR-19a were used as described in materials and method section, PrimeFlow RNA Assay. Median fluorescence index (MFI) values were used to determine expression levels of *SGK1* mRNA and miR-19a in the respective population. Gating was determined using control samples by the Fluorescence minus one (FMO) approach i.e. controls containing all markers except the one of interest were used to set gates. Gating strategy can be seen below.

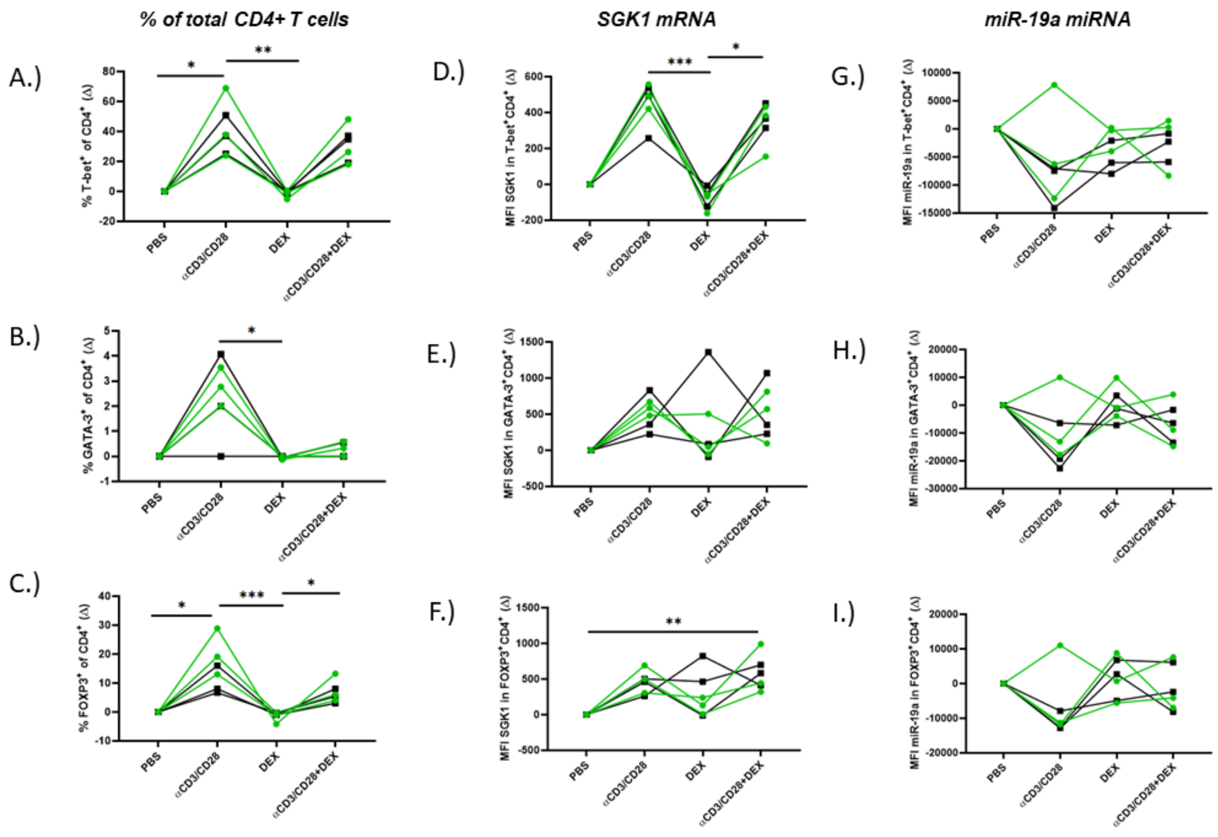

Figure S2. Examination of *SGK1* and miR-19a expression in CD4+ T cell subsets by PrimeFlow analysis. PBMCs were isolated from whole blood and treated for 48 hrs with αCD3αCD28 (αCD3/CD28), dexamethasone (DEX), αCD3αCD28 and dexamethasone (αCD3/CD28 + DEX) or medium alone (PBS) before performing PrimeFlow® RNA Assay. Percentage of CD4+ T cells that express the transcription factors T-bet (A), GATA-3 (B), and FOXP3 (C). The intensity of *SGK1* mRNA (MFI) in T-bet+ (D), GATA-3+ (E), and FOXP3+ (F) CD4+ T cells. The intensity of miR-19a (MFI) in T-bet+ (G), GATA-3+ (H), and FOXP3+ (I) CD4+ T cells. A green line indicates a individual with asthma and black line indicates a healthy individual (n = 6). Expression is presented as change from PBS control. MFI = Median fluorescent intensity. Significance was determined by Friedman ANOVA. *= $p < 0.05$; **= $p < 0.01$; ***= $p < 0.001$.

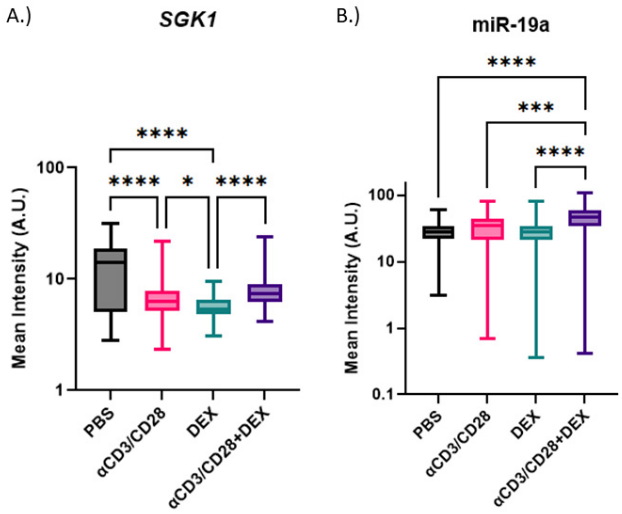

Figure S3. The mean signal intensity of *SGK1* and miR-19a change per treatment condition in CD4+ T cells. The mean intensity of *SGK1* and miR-19a was determined per cell using ImageJ. A minimum of 55 cells were included per condition. * = p<.05; *** = p<.001; **** = p < 0.0001.

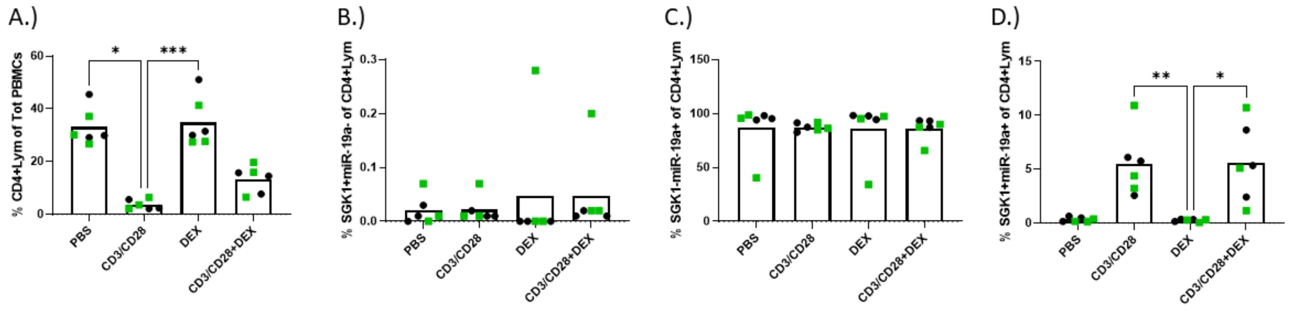

Figure S4. miR-19a, but not *SGK1*, is expressed by most CD4+ T cells. PBMCs were isolated from whole blood and treated for 48 hrs with αCD3αCD28 (CD3/CD28), dexamethasone (DEX), αCD3αCD28 and dexamethasone (CD3/CD28+DEX) or media alone (PBS) before performing PrimeFlow® RNA Assay. A) % CD4+Lym of Total PBMCs. B) % *SGK1* single positive cells of CD4+Lym. C) % miR-19a single positive cells of CD4+Lym. D) % *SGK1*+miR-19a+ double positive cells of CD4+Lym. A green square indicates an individual with asthma and a black dot a healthy subject (n = 6). * = $p < 0.05$; ** = $p < 0.01$; *** = $p < 0.001$.

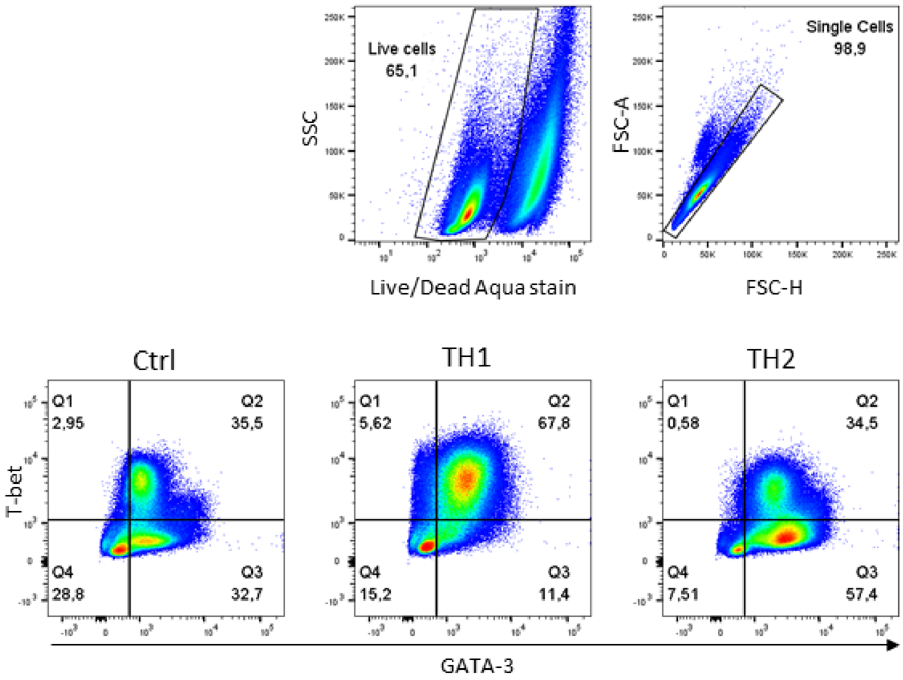

Figure S5. Gating strategy of enriched CD4+ naïve T cells polarized for 10-12 days.

Table S1: Primers used in this study

mRNA primer	*Forward sequence (5′→3′)*	*Reverse sequence (5′→3′)*
**SGK1**	AGACTACATTAATGGTGGAGAG	ATTTCAGCAGCATAGAAACG
**TBX21**	CTTTCCAAGAAACCCAGTTC	GTCAACAGATGTGTACATGG
**GATA3**	AAAATGAACGGACAGAACC	GGGGTCTGTTAATATTGTGAAG
**RORC**	TTTTGAAGGCAAATACGGTG	AGTGGGAGAAGTCAAAGATG
**FOXP3**	ACATTCCCAGAGTTCCTC	AGATCTCATTGAGTGTCCG
**NR3C1**	ACTGCTTCTCTCTTCAGTTC	GATTTTCAACCACTTCATGC
**GAPDH**	ACAGTTGCCATGTAGACC	TTGAGCACAGGGTACTTTA
miRNA primer	*miRbase name and sequence*	*Geneglobe ID (Qiagen)*
**hsa-miR-19a-3p**	MIMAT0000073:<br>5'UGUGCAAAUCUAUGCAAAACUGA	YP00205862
**hsa-miR-23a-3p**	MIMAT0000078:<br>5'AUCACAUUGCCAGGGAUUUCC	YP00204772
**hsa-miR-27b-3p**	MIMAT0000419:<br>5'UUCACAGUGGCUAAGUUCUGC	YP00205915
**hsa-miR-103a-3p**	MIMAT0000101:<br>5'AGCAGCAUUGUACAGGGCUAUGA	YP00204063
**hsa-miR-374a-5p**	MIMAT0000727:<br>5'UUAUAAUACAACCUGAUAAGUG	YP00204758

Table S2: Antibodies used in flow cytometric analysis

**Antigen (clone)**	**Format**	**Manufacturer**
CD4 (RPA-T4)	APC-H7	BD Pharmingen™, BD Biosciences
T-bet (4B10)	PE	BD Pharmingen™, BD Biosciences
T-bet (4B10)	PerCP-Cy5.5	BioLegend®
T-bet (4B10)	BV421	BioLegend®
GATA-3 (L50-823)	Pe-Cy™7	BD Pharmingen™, BD Biosciences
RORγt (Q21-559)	BV421	BD Horizon™, BD Biosciences
FOXP3 (236A/E7)	PerCP-Cy5.5	BD Pharmingen™, BD Biosciences

APC-H7, H7 conjugate of Allophycocyanin; BV421, Brilliant Violet 421; PE, phycoerythrin; PE-Cy7, Cy7 conjugate of phycoerythrin; PerCP-Cy5.5, Peridinin chlorophyll protein-Cyanine5.5.

Table S3: T cell polarization time course correlation.

miR-19a vs SGK1	3 days			5 days			7 days			10 days			All timepoints		
	R value	P value	n	R value	P value	n	R value	P value	n	R value	P value	n	R value	P value	n
**Th1 + Th2**	-0.4794	0.1148	12	0.3151	0.4913	7	0.218	0.604	8	**0.7778**	**0.0231**	8	0.2546	0.1399	35
**Th1**	-0.3846	0.4515	6	-0.3845	0.6155	4	0.1708	0.8292	4	**0.9908**	**0.0092**	4	0.2906	0.2421	18
**Th2**	**-0.8476**	**0.0331**	6	0.06402	0.9592	3	0.6038	0.3962	4	0.8722	0.1278	4	0.2196	0.397	17

Correlation between miR-19a and SGK1 were examined over a 10-day time course. n= number of subjects and **bold** values indicate significance ($p < 0.05$).
